# Arrest of root caries with an adjuvant chlorhexidine–fluoride varnish over a 12-months observation period: a QLF-analyzed, placebo-controlled, randomized, clinical trial (RCT)

**DOI:** 10.1007/s10266-021-00637-w

**Published:** 2021-07-13

**Authors:** Kyung-Jin Park, Thomas Meißner, Elena Günther, Gerhard Schmalz, Tanja Kottmann, Felix Krause, Rainer Haak, Dirk Ziebolz

**Affiliations:** 1 Department of CariologyEndodontology and Periodontology, University Leipzig, Liebigstr. 12, 04103 Leipzig, Germany; 2 Department of Prosthodontics, University Leipzig, Leipzig, Germany; 3 CRO Dr. Med. Kottmann GmbH & Co. KG, Hamm, Germany; 4 Department of Operative Dentistry, Periodontology and Preventive Dentistry, RWTH Aachen University, Aachen, Germany

**Keywords:** Root caries, Chlorhexidine–fluoride varnish, Clinical trial, Quantitative light-induced fluorescence

## Abstract

This study aimed at evaluating the effectiveness of an adjuvant chlorhexidine–fluoride varnish (Cervitec F) for prevention and arrest of root caries on elderly participants using quantitative light-induced fluorescence (QLF). 23 participants with two or three non-cavitated root carious lesions were included and assigned to three groups of different varnishes (CF: Cervitec F, P: placebo, DP: Duraphate). Agents were applied once to root surface at baseline and in follow-up after 3, 6 and 9 months. The lesions were assessed clinically and with QLF. QLF-images were analyzed regarding fluorescence loss (Δ*F*), lesion volume (Δ*Q*) and bacterial activity (Δ*R*) before (*t*_0_), after 14 days (*t*_1_), 6- (*t*_2_) and 12-months (*t*_3_). CF showed a significant difference between *t*_0_ and *t*_3_: ∆*F* (− 12.51 [15.41] vs. − 7.80 [16.72], *p* = 0.012), ∆*Q* (− 2339.97 (20,898.30) vs. − 751.82 (5725.35), *p* < 0.001), ∆*R* (23.80 [41.70] vs. 7.07 [37.50], *p* = 0.006). Independently of the varnish application, preventive care seems positively influence the root caries progress. Although within CF group the strongest effect was observed, no superiority of a specific varnish application was confirmed over a 12-months QLF observation period. Extra topical fluoride can help remineralise dentin lesions and QLF can be used as a measurement method to determine changes in the dentin lesions.

## Introduction

Dental health in the world has changed, especially in the group of senior citizens a high prevalence of periodontitis with an increasing number of remaining teeth is observed [1, 2]. Due to high periodontal disease burdens, these patients often suffer from exposed root surfaces which are particularly susceptible to root caries, resulting in one-quarter of patients having at least one carious lesion at root surfaces [2]. Especially in nursing home residences, caries prevalence is high [3]. In addition, reduced salivation (e.g. as a result of medication or dehydration in old age) promotes caries development [4]. Moreover, dental care is becoming increasingly difficult with age due to limited motoric skills or handicaps. In particular, for patients with limited general condition, root caries is difficult to treat compared to coronal caries [5]. An extension of the caries maintenance options is therefore desirable, especially in the elderly population.

In general, caries is a multifactorial disease, in which a cariogenic biofilm plays a key role [6]. Accordingly, for caries prevention, reduction of cariogenic microorganisms by mechanical and/or chemical control to regain a balanced non-pathological microflora plays a major role in therapy as well as prevention of dental caries [7]. With the reduced ability of elderly performing mechanical biofilm control [3], chemical measures are of high relevance to control the caries process. In this context, avoidance of root caries progression of initial lesions is an important preventive goal [8].

Nowadays, a multitude of different anti-caries agents is available. Numerous clinical studies have already shown the efficacy of fluoride-releasing agents to postpone demineralization and simultaneously speed up remineralization [9–11]. In particular, for the prevention and early noninvasive treatment of root caries, application of a high-concentration fluoride is more effective than the use of a standard fluoride toothpaste [8]. Besides fluoride, recent dental care products contain bacteriostatic or bactericidal substances such as chlorhexidine (CHX), enzymes, phenol derivatives, and essential oils, etc., which especially help to remedy a lack of oral hygiene via tooth brushing [12]. A systematic review and meta-analysis reported that professionally applied CHX varnish might inactivate root caries lesions or reduce their initiation [8]. Although fluoride-/chlorhexidine-containing varnishes have been introduced to the market, currently, there is little evidence of their efficacy [12, 13].

The current study aimed at investigating an ammonium fluoride-/chlorhexidine-containing varnish (Cervitec F) regarding its potential to prevent further progression of carious lesions on exposed root surfaces.

A placebo-controlled, randomized clinical trial (RCT) was set up, measuring the outcome by quantitative light-induced fluorescence (QLF), to evaluate Cervitec F as an adjuvant noninvasive treatment of root carious lesions. A placebo as well as a highly concentrated fluoride varnish (Duraphate) served as a control. The following hypotheses were put to the test: (1) the application of fluoride-/chlorhexidine-containing varnish arrests root carious lesions more effectively than placebo and (2) the effectiveness of Cervitec F is comparable to a highly concentrated fluoride varnish.

## Materials and methods

### Study design

**Fig. 1:**
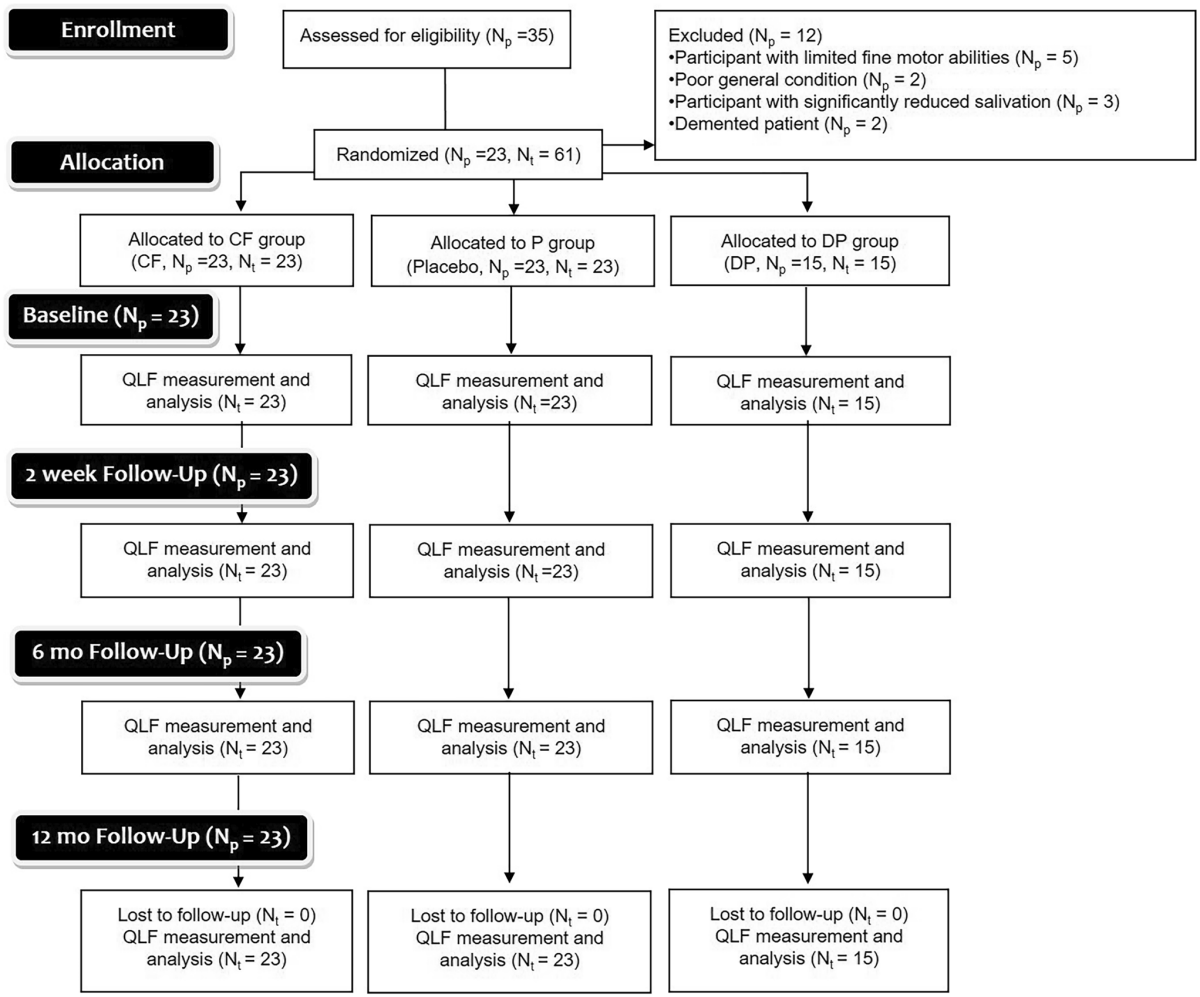
Participant flow through the randomized clinical trial (RCT) according to the CONSORT guidelines

The current study was designed as a prospective, double-blind, placebo-controlled, randomized controlled trial (RCT). Following a three-armed design, participants received one out of three application agents (incl. placebo). Figure 1 shows the flowchart according to the CONSORT guidelines.

### Study population (Fig. 1)

This study intended to detect changes in ΔF between the groups with a power of 80%. Thereby, the effect size was supposed to be 16% with a variance of 10%. At least 18 samples (teeth) were required for each group (two group design) at the significance level of 5%, without considering a normal distribution. It was aimed to include at least 23 subjects in each group to compensate potential dropouts. A total of 35 participants were screened for eligibility, 23 of which were included in the study.

The following inclusion criteria were defined:

- age between 60 and 79 years
- two or three exposed, non-adjacent root surfaces (only labial/buccal) with non-cavitated carious lesions on permanent anterior teeth or premolars regardless maxilla or mandible (score 1 = non-cavitated root caries ≤ 5 mm diameter, score 2 = non-cavitated root caries > 5 mm diameter)
  The exclusion criteria were:
- participant with limited fine motoric abilities which affect oral health procedures
- poor general condition
- participant with significantly reduced salivation (unstimulated/stimulated)
- probands with dementia
- immunosuppression/immunosuppressive drugs
- tumor disease
- Hepatitis A, B, C, TBC, HIV
- addicted patients (alcohol dependence)
- known allergy to ingredients of used agents

### Participants

The participants’ characteristics are displayed in Table 1. Age, gender and salivary parameters were comparable between groups (*p*_i_ > 0.05). The majority of lesions were scored 1, representing early caries lesions (Table 1).

**Table 1:** Patients characteristics

	Total	CF group	P group	DP group	*p* value
Number of teeth (*n* [%])	61 (100)	23 (37.7)	23 (37.7)	15 (24.6)	–
Gender (*n* [%])					
Female	27 (44)	10 (43)	10 (43)	7 (46)	*p* = 0.98
Male	34 (56)	13 (57)	13 (57)	8 (54)	
Age in years (mv ± sd)	68.25 ± 10.46	68.04 ± 10.62	68.04 ± 10.62	68.87 ± 10.70	*p* = 0.97
Visual inspection score (*n* [%])					
Score 1	45 (74)	18 (78)	16 (70)	11 (73)	*p* = 0.80
Score 2	16 (26)	5 (22)	7 (30)	4 (27)	
Salivary flow rate (*n* = 53; ml/5 min; (mv ± sd)					
Un-stimulated	1.00 ± 0.74	0.98 ± 0.75	0.98 ± 0.75	1.05 ± 0.76	*p* = 0.95
Stimulated	5.18 ± 2.95	5.15 ± 3.02	5.15 ± 3.02	5.26 ± 2.96	*p* = 0.99
Reduced salivary flow (*n* [%])	0	0	0	0	–
Salivary buffer capacity (*n* = 53; *n* [%])*					
Low	3 (6)	1 (4)	1 (4)	1 (7)	*p* = 0.97
Medium	17 (32)	6 (26)	6 (26)	5 (33)	
High	33 (62)	13 (70)	13 (70)	7 (60)	

*mv* mean value, *sd* standard deviation

*CRT buffer Test, IvoclarVivadent, Schaan Liechtenstein

### Test material and group allocation

An ammonium fluoride-/chlorhexidine-containing varnish (group CF; Cervitec F; Ivoclar Vivadent AG, Schaan, Lichtenstein) was compared with a placebo varnish (group P; Ivoclar Vivadent AG) which was based on the essential composition of Cervitec F, excluding ammonium fluoride, chlorhexidine (CHX) and cetylpyridinium chloride (CPC). In case of presence of a third tooth with a root caries lesion, a high fluoride varnish (group DP; Duraphat; Colgate Oral Pharmaceutical, Inc, Canton MA, USA) was applied as a control. Table 2 indicates the compositions of the materials. The affected teeth of each participant were randomly assigned to one of the three groups. In case of participants with only two teeth included, these were randomly allocated to the groups CF and P, respectively. The randomization and group allocation procedure was performed by an independent person, which did not participate in clinical examination or treatment. Table 3 shows the distribution of included teeth for the different groups.

**Table 2:** Allocation of groups and procedure for application of used materials according to manufacturer’s recommendations

Material	Composition	Application
CF group	Alcohol/aqua (80–90 wt.-%) Vinylacetat/crotonates copolymer Cetylpyridinium chloride (0.5%) Chlorhexidine diacetate (0.3%) Ammonium fluoride (fluoride content: 1400 ppm) aroma saccharin	Three-monthly application of Cervitec F 1. Isolation of the application area with cotton rolls 2. Applying the varnish once in a thin layer using a brush 3. Drying varnish for 1 min 4. Removing the cotton rolls Application of fluoride-containing toothpaste (twice per day, for 2 min.; 1250 ppm, Dentagard, Colgate Oral Pharmaceutical, Inc, Canton MA, USA)
Cervitec F (Ivoclar Vivadent AG, Schaan, Lichtstein)		
P group	Alcohol/aqua (80–90 wt.-%) Vinyl acetate/crotonates copolymer Aroma Saccharin	Three-monthly application of placebo 1. Isolation of the application area with cotton rolls Applying the agent once in a thin layer using a brush Drying varnish for 1 min. 4. Removing the cotton rolls Application of fluoride-containing toothpaste (twice per day, for 2 min.; 1250 ppm, Dentagard, Colgate)
Placebo (Ivoclar Vivadent AG, Schaan, Lichtstein)		
DP group	Colophonium Ethanol Sodium fluoride (fluoride content: 22,600 ppm) saccharin Isoamyl acetate Mastic Shellac Bleached wax	Three-monthly application of Duraphat 1. Isolation of the application area with cotton rolls 2. Applying the varnish once in a thin layer using cotton swabs 3. Drying varnish for 1 min 4. Removing the cotton rolls Application of fluoride-containing toothpaste (twice per day, for 2 min.; 1250 ppm, Dentagard, Colgate)
Duraphat (Colgate Oral Pharmaceutical, Inc, Canton MA, USA)

**Table 3:** Distribution of included teeth between groups

	Total (*n* = 61)	CF group (*n* = 23)	P group (*n* = 23)	DP group (*n* = 15)
Anterior maxilla	19	9	8	2
Premolar maxilla	6	1	3	2
Anterior mandible	24	8	8	8
Premolar mandible	12	5	4	3

### Outcome parameter

Quantitative light-induced fluorescence (QLF, QRayCam v.1.00, serial no.: 15090005, Software C3 v 1.26 Inspektor Research Systems, Amsterdam, Netherlands) was applied to assess the effect of the applied varnishes on the treated root caries. As examination parameters, fluorescence loss/demineralization states (Δ*F*, %), lesion volume/area (Δ*Q*, mm^2^ x %) and increase of red fluorescence (Δ*R*, %) were measured at baseline (*t*_0_), after 2 weeks (*t*_1_), 6 months (*t*_2_) and 12 months (*t*_3_).

Validation of the QLF method [14]: For the validation of the QLF method, 46 exposed non-cavitated root surfaces of 12 participants were investigated. The regions of interest (ROIs) were classified into three groups by visual inspection: sound (0), lesion ≤ 5 mm (1), lesion > 5 mm in diameter (2). Three examiners imaged every ROI three times using QLF (QRayCam) and measured fluorescence loss (Δ*F*), lesion volume (Δ*Q*) and increase of red fluorescence (Δ*R*). The threshold of the edge of the lesion was 95%. The intra- and interexaminer reproducibilities were calculated (intraclass correlation coefficient, ICC). The correlation between the lateral extent of non-cavitated root caries lesions and QLF-analysis was determined.

### Study flow (Fig. 2)

**Fig. 2:**
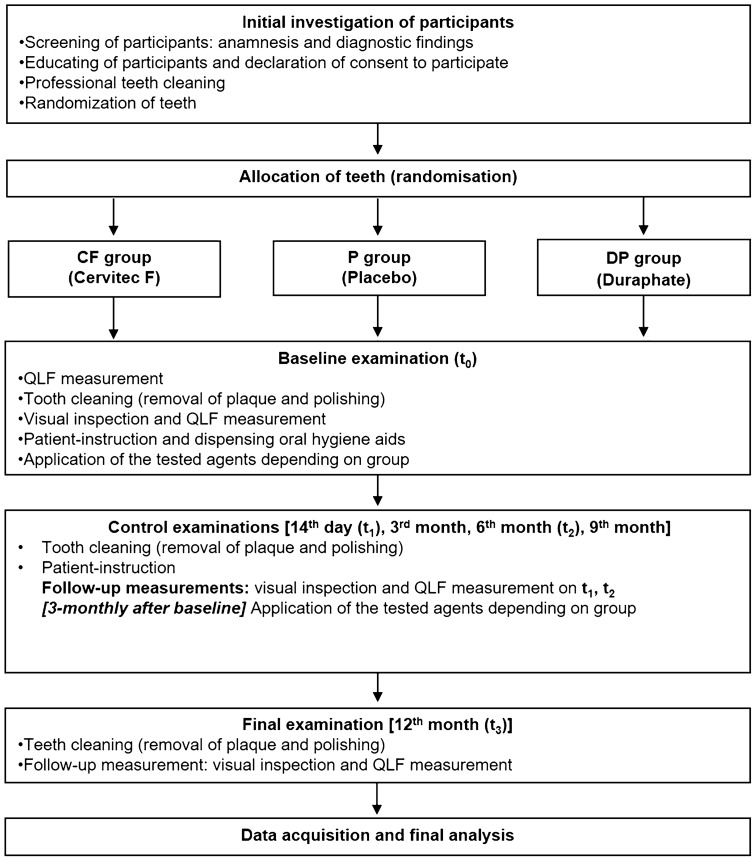
Workflow

The investigations were carried out from April 2017 to August 2018. After the initial check of the eligibility by in- and exclusion criteria, participants were informed about the study course and gave written informed consent. Afterwards, they received a professional tooth cleaning to ensure comparable conditions at the beginning of the investigation. All examinations were performed blind (with regards to agents applied) under standardized conditions by an experienced, calibrated (kappa > 0.8) and. Figure 2 summarizes the flow of the study.

At baseline (*t*_0_), QLF measurement was performed at first. After removal of plaque with a disposable polishing cup without polish paste (Prophy Angle Lavender Soft Cup, LOT: 20,170,109, Dentsply Sirona, York, USA) visual inspection (score 1 and 2 according to the inclusion criteria) and QLF measurement were performed. Participants and examiner used protective eyewear. During this appointment, participants-received oral hygiene aids for dental home care (toothbrush: SUNSTAR GUM ActiVital, Sunstar Deutschland GmbH Kriftel, Germany; toothpaste: Dentagard, Colgate Oral Pharmaceutical, Inc, Canton MA, USA; renewed every 3 months) and were informed and instructed about tooth brushing (twice per day for 2 min). The participants used these oral hygiene aids throughout the entire observation period and no further chemical control of biofilm was performed. At the end of the baseline appointment, the two or three different agents were applied following the group allocation (Table 1). Application was repeated 3-monthly (3, 6 and 9 months after baseline). Participants should not have eaten and drunk 1 h before and after varnish application.

After the baseline QLF examination (*t*_0_), further control examinations were carried out in the 2nd week (*t*_1_), 3rd month, 6th month (*t*_2_) 9th month and 12th month (*t*_3_). During each appointment, the teeth were cleaned (biofilm removal and polishing), participants were reinstructed in oral hygiene procedures (except for the final visit), and QLF measurement was performed on the test teeth in accordance to baseline examination. After the final examination (*t*_3_), the QLF images were evaluated under standardized conditions (artificial light, no window, air conditioner set to 23 °C, Monitor: Dell LCD monitor, model no. U2212HMc, signal resolution: 1920 × 1080, refresh rate: 60 Hz, bit depth: 8-bit, color format: RGB, color space: SDR) by a calibrated and blinded investigator. Since some parameters (e.g. Δ*Q*) were stated in pixels they were converted into μm by using reference patches of a defined size of 2 × 2 mm and an image analysis software (ImageJ2 v. 1.52a). A direct pixel based comparison of ΔQ is impossible since the photographic distance and thus the image scale varied due to the free-hand image acquisition technique.

### Statistical analysis

All statistical analyses were executed using SPSS for Windows, Version 24.0 (SPSS Inc., U.S.A.). To test the variables for normal distribution, Shapiro–Wilk-test was applied. The Levene-test was used to test samples for their homogeneity of variance, which showed a sufficient similarity in the allocation of the samples that allowed execution of univariate analysis. For more than two dependent, normal distributed samples, the general linear model was applied, while Friedman-test was used in case of non-normal distribution. In case of significance, post-hoc testing using Least Significant Difference as well as Bonferroni test was applied. The significance level was set at *p* ≤ 0.05.

## Results

### QLF assessment: method validation [14]

Intra- and interexaminer reproducibility (ICC) was 0.98 and 0.95 for Δ*F*, 0.94 and 0.91 for Δ*Q*, 0.79 and 0.95 for Δ*R*, respectively. A significant correlation was observed between the lateral extent of caries lesions and Δ*F* (*ρ* = − 0.53, *p* < 0.01). For sound surface (score 0) the median Δ*F* value was 0% (IQR = 0), for score 1a = − 10% (IQR = 12) and for score 1b = -23% (IQR = 11).

### QLF results within groups

Within CF and P group, a significant effect of time (*t*_0_—*t*_3_) was detectable for each QLF parameter, including ∆*F*, ∆*Q* and ∆*R* (*p*_i_ < 0.05; Table 4); in the DP group an effect of time (*t*_0_—*t*_3_) was found only for ∆*R* (Table 4). However, in post-hoc analysis only CF group showed a significant difference between *t*_0_ and *t*_3_ for ∆*F* (− 12.51 [15.41] vs. − 7.80 [16.72], *p* = 0.012), ∆*Q* (− 2339.97 (20,898.30) vs. − 751.82 (5725.35), *p* < 0.001) as well as ∆*R* (23.80 [41.70] vs. 7.07 [37.50], *p* = 0.006; Table 4). Furthermore, for CF a statistically significant difference of ∆*Q* was detectable after 6 months (*t*_0_—*t*_2_: − 2339.97 (20,898.30) vs. − 417.13 (4329.76), *p* = 0.002; Table 4).

**Table 4:** Results for Δ*F*, Δ*Q* and ∆*R* depending on examination time of groups (median [IQR])

	CF group	P group	DP group
Δ*F* (%)			
Baseline	− 12.51 (15.41)	− 10.35 (10.06)	− 11.04 (19.45)
*T*_1_	− 11.25 (10.92)	− 8.93 (11.99)	− 8.95 (24.90)
*T*_2_	− 9.91(15.06)	− 7.62 (12.82)	− 8.53 (15.28)
*T*_3_	− 7.80 (16.72)*	− 9.17 (8.65)	− 7.81 (20.00)
*p* value	0.008	0.037	0.066
Δ*Q* (% µm^2^)			
Baseline	− 2339.97 (20,898.30)	− 1382,91 (18,459,39)	− 1721.30 (4869.73)
*T*_1_	− 1851.40 (15,683.66)	− 1184.65 (10,424,66)	− 1796.66 (21,060.01)
*T*_2_	− 417.13 (4329.76)*	− 1273.42 (10,354,86)	− 922.72 (15,257.73)
*T*_3_	− 751.82 (5725.35)*	− 1301.23 (15,230,32)	− 989.45 (11,894.92)
*p* value	< 0.001	0.015	0.113
Δ*R* (%)			
Baseline	23.80 (41.70)	25.00 (45.43)	16.27 (36.37)
*T*_1_	27.47 (32.44)	25.57 (35.70)	22.27 (46.83)
*T*_2_	17.97 (36.83)	23.47 (36.83)	24.70 (39.20)
*T*_3_	7.07 (37.50)*	24.83 (33.03)	7.37 (43.17)
*p* value	< 0.001	0.001	0.006

*IQR* inter-quartile-range

*Significant finding in post-hoc analysis in comparison to baseline (*p* < 0.05)

### Comparison of differences in QLF between groups

The QLF differences of ∆F, ∆Q, and ∆R between groups after 14 days (*t*_0_—*t*_1_), 6 months (*t*_0_—*t*_2_) and 12 months (*t*_0_—*t*_3_) are given in Table 5. Although different changes of the investigated parameters in the examined groups (CF, P, and DP) are present, no statistically significant group effect was detectable (*p*_i_ > 0.05; Table 5).

**Table 5:** Comparison of the three study parameters (median Δ*F*, Δ*Q* und Δ*R*) between groups from Baseline to *t*_1_, *t*_2_ and *t*_3_

Parameter/time point	CF group	P group	DP group	*p* value
Δ*F* (%)				
*T*_1_	− 0.97	0	0	0.552
*T*_2_	− 3.34	− 2.19	0	0.324
*T*_3_	− 3.41	− 1.46	− 2.25	0.320
Δ*Q* (% µm^2^)				
*T*_1_	− 1314.50	0	0	0.178
*T*_2_	− 1692.09	− 482.25	− 194.80	0.535
*T*_3_	− 1588.15	− 731.24	− 194.80	0.903
Δ*R* (%)				
*T*_1_	0	− 0.23	0	0.574
*T*_2_	4.40	1.77	0	0.185
*T*_3_	7.77	0.50	7.53	0.115

### Differences depending on lesion score

Significant differences for ∆*F*, ∆*Q*, and ∆*R* findings were present between lesion score 1 and 2 independent of the group assignment (*p*_i_ < 0.01, Table 6). Thereby, a group interaction was found for ∆*Q* at *t*_1_ (*p* = 0.016) and for ∆*R* at *t*_1_ (*p* = 0.038), which could not be confirmed in post-hoc testing between groups (*p*_i_ > 0.05, Table 6).

**Table 6:** Comparison of the difference of measured parameters (median ΔF, ΔQ und ΔR) between groups from Baseline to *t*_1_, *t*_2_ and *t*_3_ under consideration of the visual inspection score

Parameter/time point	CF group		P group		DP group		*p* value	*p* value group comparison		
	Score 1 (*n* = 18)	Score 2 (*n* = 5)	Score 1 (*n* = 16)	Score 2 (*n* = 7)	Score 1 (*n* = 11)	Score 2 (*n* = 4)		CF vs. P	CF vs. DP	P vs. DP
ΔF (%)										
*T*_1_	− 0.70	− 2.47	0	− 1.57	− 0.76	4.69	0.069	–	–	–
*T*_2_	− 3.23	− 3.57	− 1.60	− 2.41	− 1.75	0.71	0.652	–	–	–
*T*_3_	− 2.79	− 4.99	− 0.80	− 2.49	− 2.25	− 1.56	0.134	–	–	–
ΔQ (% µm^2^)										
*T*_1_	− 786.15	− 8967.47	32.27	− 6143.83	0	7318.90	0.016	0.624	0.057	0.138
*T*_2_	− 1652.52	− 11,560.54	− 344.30	− 3133.15	− 194.80	− 4864.97	0.889	–	–	–
*T*_3_	− 1428.61	− 13,390.54	− 476.63	− 5218.28	− 194.80	3956.53	0.785	–	–	–
Δ*R* (%)										
*T*_1_	0	6.07	− 0.12	− 2.83	0.57	− 16.83	0.038	0.269	0.732	0.520
*T*_2_	4.78	3.63	1.65	4.23	0	− 7.33	0.773	–	–	–
*T*_3_	6.30	11.17	0.10	8.17	7.53	24.72	0.417	–	–	–

## Discussion

All groups showed caries-preventive effects. The CF group in particular showed pronounced and statistically significant effects for all parameters after 12 months. Moreover, the lesion volume Δ*Q* was significantly reduced after 6 months of observation (*t*_0_—*t*_2_). Comparing the differences of the fluorescence parameters, no group effect was found in total as well as after subdivision into the different lesion scores (score 1 or 2).

Pretty et al. concluded in their studies that QLF is a valid tool for root caries assessment based on their in vitro evaluation of the method’s ability to reflect de- and remineralization processes [15]. More recent clinical studies confirmed that this method is able to estimate the demineralization of root carious lesions as well as biofilm accumulation on these lesions [16, 17]. Based on these findings, QLF was chosen for the detection and quantification of mineralization processes and various demineralization states at root surfaces as well as their longitudinal monitoring in the current study.

In the current study, QLF served as a non-invasive technique to measure the effect of applied varnishes and delivered the main outcome of the examination. This procedure is on the one hand based on the auto-fluorescence of tooth substance (Δ*F*, Δ*Q*), which provides information about potential demineralization caused by caries [18]. On the other hand, red fluorescence signals of bacterial degradation products like porphyrins are provided (Δ*R*).

Recent review articles have already discussed suitable prevention and management of root caries lesions [8, 19]. The high importance of this issue is underlined by the high prevalence of root caries especially in the elderly population [2] and the occurrence of carious lesions at the root surface after periodontal therapy [20]. The most suitable therapeutic strategy is discussed controversially. Several studies described that a higher fluoride concentration is necessary for prevention and control of root caries compared to coronal caries [19, 21, 22]. A meta-analysis reported that daily application of 5000 ppm fluoride-containing toothpaste is more effective in reducing the activity of root carious lesions than a toothpaste containing 1100–1450 ppm fluoride [8]. Ekstrand et al. concluded in their study that adding Duraphat varnish (22,600 ppm) enables control of root caries [23].

While these studies underlined the preventive effect of high concentration fluoride on root caries lesions, the application of chlorhexidine as an anti-cariogenic substance is a further approach [8]. It was reported that neither CHX-rinses nor -varnishes had a preventive effect on root caries [12]

Meanwhile other studies indicated that daily rinse with 0.12% CHX inhibited the growth of cariogenic bacteria on the root surface [24] and that CHX varnish reduced root caries significantly [25]. Moreover, CHX varnish was especially recommended for root caries on patients in need of special care [26]. In addition, a combined application of CHX and fluoride or mixtures of CHX with other substances (such as thymol or fluoride) exhibited an anti-cariogenic effect on the root surface [12, 27–31]. While the high fluoride concentrated varnish (DP) in the current study did not show statistically significant differences between the measurements at the different observation points, CF as CHX/fluoride combination showed these significances for long term results over 12 months (comparison *t*_0_–*t*_3_). The application of CHX varnish every 3–4 months to prevent root caries has been recommended previously [32]. The unchanged state between baseline and 14th day in the CF group might suggest that repeated application and a certain time period would be necessary to achieve relevant remineralization and reduction of the lesion reflected by QLF measurement. The penetration of self-curing varnishes like CF into the dentin tubules has already been suggested as a potential mechanism leading to their long-term effect [33].

Moreover, as carious lesions are multifactorial and associated with a cariogenic biofilm [6], the sole remineralization with highly concentrated fluoride might be less effective than a combination with antimicrobial substances, which could explain the study’s findings regarding CF. In addition to this attempt at explanation, fluoride has antimicrobial effects itself [34], putting the biofilm modulation effect of CHX compared to a highly concentrated fluoride varnish into question. For all parameters, no significant difference between groups was observed over the full timeframe of the study. Although CF was the only application with a significant beneficial effect within the group, a statistical superiority if directly compared to the other applications is not derivable. In this respect, the absence of root caries progression in the placebo group is remarkable and is probably an effect of the tri-monthly performed tooth cleaning. Accordingly, the positive effect of the application of CF must be seen as a supplementary intervention to tooth cleaning.

This current study was a prospective, double-blind, placebo-controlled, randomized controlled trial, what is recommended for clinical trials to obtain robust results. The inclusion of subject groups with a pre-determined number of participants constant over the observational period is a further strength. However, several limitations exist: The location of the teeth (maxilla or mandible) was not taken into account. Additional investigation of cariogenic components such as socio-economic background, caries risk and plaque index would have helped to understand if any changes in oral health parameters in the 12 months of the study had occurred. Furthermore, it might be conceivable that all participants increased their oral hygiene behavior because of participation in a clinical study, what might blur an effect of the intervention (Hawthorne-effect). The DP group in the current study had a different sample size compared to the other groups. The professional tooth cleaning performed at different time points could have had an effect on the root caries that might have limited the additional benefit of the selected applications. While this was necessary to ensure comparable conditions for the participants, it is not a very realistic situation, especially for nursing home residents. The agents investigated were applied to different teeth of the same participant during one session. While this could affect the study results this approach was chosen to ensure the same intraoral condition for the objective comparison of the agents; different environmental and subject-specific factors might have influenced the root caries formation and progression in the different participants, making fully equal conditions impossible. Furthermore, the applied methodology seems to be very sensitive and could have been influenced by these conditions. Especially the small sample size of each lesion score, might have led to missing significance due to too low discriminatory power. Therefore a suggestion for future studies would be to increase the sample size and improve the selection of dental organs by anatomical field. Despite the listed limitations, the current study delivered insights into the management of root caries in the elderly population.

Independently of the varnish application, preventive care seems to positively influence the root caries progress over 12 months. Based on this study it was found that extra topical fluoride can help remineralise dentin lesions and QLF can be used as a measurement method to determine changes in the dentin lesions. More research on a larger scale is necessary to confirm the findings.
